# A Sequential Statistical Approach towards an Optimized Production of a Broad Spectrum Bacteriocin Substance from a Soil Bacterium *Bacillus* sp. YAS 1 Strain

**DOI:** 10.1155/2014/396304

**Published:** 2014-12-29

**Authors:** Amira M. Embaby, Yasmin Heshmat, Ahmed Hussein, Heba S. Marey

**Affiliations:** ^1^Department of Biotechnology, Institute of Graduate Studies and Research, University of Alexandria, 163 Horreya Avenue, P.O. Box 832, Chatby 21526, Egypt; ^2^Department of Environmental Studies, Institute of Graduate Studies and Research, University of Alexandria, P.O. Box 832, Chatby 21526, Egypt; ^3^University of Alberta, Department of Civil and Environmental Engineering, Edmonton, AB, Canada

## Abstract

Bacteriocins, ribosomally synthesized antimicrobial peptides, display potential applications in agriculture, medicine, and industry. The present study highlights integral statistical optimization and partial characterization of a bacteriocin substance from a soil bacterium taxonomically affiliated as *Bacillus* sp. YAS 1 after biochemical and molecular identifications. A sequential statistical approach (Plackett-Burman and Box-Behnken) was employed to optimize bacteriocin (BAC YAS 1) production. Using optimal levels of three key determinants (yeast extract (0.48% (w/v), incubation time (62 hrs), and agitation speed (207 rpm)) in peptone yeast beef based production medium resulted in 1.6-fold enhancement in BAC YAS 1 level (470 AU/mL arbitrary units against *Erwinia amylovora*). BAC YAS 1 showed activity over a wide range of pH (1–13) and temperature (45–80°C). A wide spectrum antimicrobial activity of BAC YAS 1 against the human pathogens (*Clostridium perfringens*, *Staphylococcus epidermidis*, *Campylobacter jejuni*, *Enterobacter aerogenes*, *Enterococcus* sp., *Proteus* sp., *Klebsiella* sp., and *Salmonella typhimurium*), the plant pathogen (*E. amylovora*), and the food spoiler (*Listeria innocua*) was demonstrated. On top and above, BAC YAS 1 showed no antimicrobial activity towards lactic acid bacteria (*Lactobacillus bulgaricus*, *L. casei*, *L. lactis*, and *L. reuteri*). Promising characteristics of BAC YAS 1 prompt its commercialization for efficient utilization in several industries.

## 1. Introduction

Ribosomally synthesized antimicrobial peptides (AMPs) are produced by prokaryotes (e.g., Gram-negative bacteria and Gram-positive bacteria) and eukaryotes (e.g., plant and a wide variety of animals both invertebrates and vertebrates). In unicellular microorganisms those AMPs produced by bacteria are designated as bacteriocins [1–4]. The present research paper focuses on AMPs of bacterial origin (bacteriocins).

Bacteriocins can antagonize the growth of closely related species (narrow spectrum) or across genera (broad spectrum). Their usage as potential weapons against human/animal/plant pathogens and food spoiler microorganisms has attracted the scientific attention in recent years [1, 5–8].

Classification of bacteriocins is not an easy task because continuous alterations in this classification exist. These alterations are mainly derived from heterogeneity in bacteriocins pertaining to their structures, amino acid sequences, and mechanisms of action. Although bacteriocins are a heterogonous group, they could be categorized into two main classes: class I (lanthionines with unusual amino acids) and class II (non-lanthionine containing unmodified bacteriocins). Lantibiotics (lanthionines containing posttranslationally modified bacteriocins) contain unusual amino acids such as lanthionine (Lan), methyl lanthionine (MeLan), dehydroalanine (Dha), dehydrobutyrine (Dhb), or D-alanine (D-Ala), whilst non-lanthionines containing bacteriocins do not undergo extensive posttranslational modifications. So far, a confined number of bacteriocins have been studied [8–11].

Diverse mechanisms describing the bacteriocin mode of action were outlined but the cell membrane still remains the main target of these AMPs. The net positive charges of bacteriocins stimulate preferential binding of bacteriocins to the negatively charged bacterial cell membranes resulting in creation of multiple pores in the cell membranes [12–14]. In the context of bacteriocins as an adequate alternative to antibiotics against multidrug resistant pathogens along with a continuous urgent need for developing new antimicrobial agents, bacteriocins represent a promising inexhaustible source of new antibiotics [15, 16].

The objective of the present study is to isolate a bacteriocin producing bacterium with a wide spectrum antimicrobial property. The aim is extended to optimize the production of the produced bacteriocin through a sequential statistical approach. To the best of our knowledge, there exists a plethora of literature concerning characterization of bacteriocins but there exists a few reports pertaining to highlighting key determinants controlling the production of bacteriocins by their producers.

## 2. Materials and Methods

### 2.1. Chemicals

Majority of bacteriological media used in this study were purchased from Oxoid Co., UK, unless otherwise stated. Proteinase K and Dream Taq Green Master Mix 2X were purchased from Thermo Scientific Co., USA. Isopropanol, acetone, acetonitrile, toluene, methyl alcohol, and ethyl alcohol were purchased from Sigma, Co., USA. Lysozyme and agarose were purchased from Bio Basic Inc., Canada. DNA ladder (100 bp DNA Ladder H3 RTU (Ready-to-Use) was purchased from GeneDirex, Inc., Taiwan. Universal primers specific for* 16S rDNA gene* were synthesized by Bioneer Co., Korea.

### 2.2. Indicator Strains

*Erwinia amylovora*, pear fire blight pathogen, and lactic acid bacterial strains (*Lactobacillus bulgaricus*,* L. casei*,* L*.* lactis,* and* L. reuteri*) were obtained from the bacterial culture collection of the Department of Plant Pathology and the Department of Dairy Products, respectively, Faculty of Agriculture, University of Alexandria, Egypt. The human pathogens indicator strains used in this study were* Salmonella typhimurium* NCTC 12023/ATCC 14028,* Clostridium perfringens* NCTC 8237/ATCC 13124,* Staphylococcus aureus* NCTC 12981/ATCC 25923,* S. epidermidis* NCTC 13360/ATCC 12228,* Escherichia coli* NCTC 12241/ATCC 25922,* Shigella boydii* NCTC/ATCC 9207,* Campylobacter jejuni* NCTC 11322/ATCC 29428,* Enterococcus faecalis* NCTC 12697/ATCC29212,* Enterobacter aerogenes* NCTC10006/ATCC13043,* Candida albicans NCPF* 3179/ATCC10231, and* Vibrio parahaemolyticus* NCTC 3178. Other human clinical pathogens such as* Proteus* sp.,* Klebsiella* sp.,* Enterobacter* sp.,* Citrobacter* sp.,* Enterococcus* sp.,* S. typhi*,* Pseudomonas aeruginosa*,* Acinetobacter* sp.,* E. coli*, and* S. paratyphi* were obtained from the Department of Microbiology, Faculty of Medicine, University of Alexandria, Egypt.* Candida tropicalis* was obtained from the Department of Microbiology, Medical Research Institute, University of Alexandria, Egypt. The food spoiler indicator strain was* Listeria innocua* NCTC 11288/ATCC 33090. Other environmental microbial strains included* B. cereus*,* B. licheniformis* SHG2,* B. licheniformis* SHG6,* B. licheniformis* SHG10,* Bacillus subtilis* NCTC 10400/ATCC 6633,* B. subtilis* AS1,* B. subtilis*,* B. subtilis* EMBLAKE,* Bacillus* sp. Ash2,* P. aeruginosa* strain EGYII,* Aspergillus brasiliensis* NCPF2275/ATCC16404, and* Saccharomyces cerevisiae* NCPF 3178. All indicator bacterial strains were preserved in 15% glycerol stocks at −80°C. However, fungal and yeast strains were preserved in agar slopes at 4°C.

### 2.3. Media

Peptone yeast beef medium (PYB in g/L: 10 g peptone, 5 g yeast extract and 3 g beef extract) was used as a bacteriocin core production medium.* E. amylovora* was grown on PYB supplemented with 2% sucrose. The indicator strains (*S. typhimurium*,* S. paratyphi*,* E. coli*,* P. aeruginosa*,* Klebsiella* sp.,* Enterobacter* sp.,* Citrobacter* sp., and* Proteus* sp.) were grown on BHI (Brain Heart Infusion medium, Oxoid CM 1135). All strains of lactic acid bacteria were grown on MRS (De Man Rogosa Sharpe, Oxoid CM0361).* C. albicans* and* C. tropicalis* were grown on SAB Dextrose Agar medium (Sabouraud Dextrose Agar, Oxoid, CM004).* S. aureus* and* S. epidermidis* were grown on MSA (Mannitol Salt Agar, Oxoid CM 0085).* C. perfringens* was grown on TSC (Tryptose Sulphite Cycloserine Agar, LAB M194).* A. brasiliensis* and* S*.* cerevisiae* were grown on Dichloran Glycerol medium (HIMEDIA, M1129-500G).* L. innocua* was grown Brilliance Listeria Agar Base (Oxoid, CM1080). All strains belonging to the genus* Bacillus *were grown on PYB medium.* V. parahaemolyticus* was grown on thiosulphate citrate bile salts sucrose agar (TCBS medium, LAB M96).* E. faecalis* was grown on KF Streptococcus Agar Base (Oxoid CM0701). Sporulation medium (1XSG) [17] in g/L: 6 g beef extract, 10 g peptone, 2 g KCl, 1 g glucose plus 1 mM CaCl_2_, 1 mM FeSO_4_, 1 mM MnCl_2_, and 2 mM MgSO_4_) was used during the course of studying the most promising isolate under the electron microscope. MYP (Mannitol egg yolk/phenol red; CM0929, Oxoid, Co.) medium consisted of 1.0 g meat extract, 10 g peptone, 10 g NaCl, 10 g mannitol, 0.025 g per liter plus supplements of polymyxin, and egg yolk suspension [18]. Sheep blood agar was obtained from the Department of Microbiology, Faculty of Medicine, University of Alexandria, Egypt.

### 2.4. Isolation of Bacteriocin Producing Bacteria

Different samples including soils, leaves, and fruits were collected from different sites in Borg El-Arab in Alexandria city, Egypt. The candidate sites were naturally contaminated with the pear firelight pathogen* E. amylovora*. One gram of each sample separately was used to inoculate 20 mL of PYB broth in 250 mL Erlenmeyer flask for enrichment. The inoculated broth was incubated for an overnight at 37°C with agitation speed of 150 rpm (New Brunswick Incubator Shaker, USA). Serial dilutions were prepared from each enriched culture. One hundred microliters of an appropriate dilution from each culture was selected to be spread on the surface of PYB agar plates. Next day, raised single colonies were purified by streaking technique. Bacteriocin capability of each bacterial isolate was checked via tooth picking technique as a primary screening. Briefly, PYB agar was inoculated with 1% of an overnight culture of the indicator strain* E. amylovora*. Then the inoculated agar was poured into plates. After solidification, the agar plates were stabbed using sterile tooth picks carrying the previously isolated single colonies to assess their efficiency for bacteriocin production. After that, these stabbed inoculated agar plates were incubated at 37°C for an overnight (JSGI-100T, Incubator, Korea). Halo zones of the growth of the indicator strain around the stabbing points were taken as a tentative indicative for bacteriocin production. Bacterial isolates having antimicrobial effect against* E. amylovora* were further subjected to a secondary screening via well cut diffusion assay as mentioned below in Section 2.9. The most potent bacteriocin producer was selected as a good candidate for a further detailed study in order to integrally optimize and partially characterize the produced bacteriocin.

### 2.5. Identification of the Bacteriocin Producing Isolate

#### 2.5.1. Morphological and Biochemical Identification

The most potent bacteriocin producing isolate was identified morphologically and biochemically according to Bergey's manual of determinative bacteriology [19]. Cell morphological features of the bacteriocin producer were studied under light microscope and electron microscope (transmission electron microscope (TEM) and scanning electron microscope (SEM)).

#### 2.5.2. Molecular Identification

Amplification of* 16S rDNA gene* was carried out to identify the bacteriocin producer isolate on a molecular level. Genomic DNA of the bacteriocin producer was extracted according to a procedure reported previously by Sambrook et al. [20]. The gene encoding* 16S rDNA* was amplified from the genomic DNA by PCR using previously described universal primers F8-27 (5′-AGAGTTTGATCCTGGCTCAG-3′) and R1510-1492 (5′-GGTTACCTTGTTACGACTT-3′) of* Escherichia coli 16S rDNA gene* [21]. The reaction mixture (50 *μ*L) contained 3 *μ*L (30 ng genomic DNA), 25 *μ*L of Dream Taq Green Master Mix 2X (Thermo Scientific Co., USA), 1.5 *μ*L (15 pmol) of each forward and reverse primer, and 19 *μ*L of nuclease free water. Thermocycler primus 25 (peQlab, Erlangen, Germany) was used in this study. PCR conditions consisted of an initial denaturation at 95°C for 5 min, 30 cycles: each cycle consisted of denaturation at 94°C for 30 s, annealing at 55°C for 45 s, extension at 72°C for 1.5 min, and a final extension at 72°C for 10 min. The amplified PCR product was analyzed via agarose gel electrophoresis. After that, the PCR product was purified using Wizard SV Gel and PCR Clean-Up kit, Promega Co., USA. Then, the purified PCR product was sequenced directly as sequencing templates using the abovementioned primers. Sequencing was carried out at Eurofin DNA Co., Luxembourg. The obtained nucleotide sequence was edited and analyzed by Geneious R8 software. A partial nucleotide sequence (1331 bases) was used to search the international nucleotide databases (e.g., GenBank, EMBL, DDBJ, etc.) through BLASTN (Basic Local Alignment Search Tool) of NCBI (National Center for Biotechnology Information) to determine the relative phylogenetic position of the bacterial isolate of inquiry. Moreover, the phylogenetic tree depicting the phylogenetic affiliation of the isolate of inquiry was constructed via Geneious R8 software.

### 2.6. Inoculum Preparation

An overnight single colony of the most potent bacteriocin producer from PYB agar plate was picked to inoculate 20 mL of PYB broth in 250 mL Erlenmeyer flask. The inoculated broth was incubated for 18 hrs at 37°C with agitation speed at 150 rpm (New Brunswick Incubator Shaker, USA). One mL of this culture (8.0 × 10^3^ CFU/mL) was used to inoculate the bacteriocin production medium unless otherwise stated.

### 2.7. Optimizing Bacteriocin Productivity

The most potent bacteriocin producing bacterial isolate was selected to conduct an integral statistical optimization study for the produced bacteriocin. A sequential statistical approach (Plackett-Burman design followed by response surface methodology) was employed in this study in order to optimize the production of bacteriocin.

#### 2.7.1. Plackett-Burman Design (PBD)

PBD is a two-factorial design created by Plackett and Burman [22]. It allows studying of *N*-factors through at least *N* + 1 experiments assuming that the interactions among the studied factors are negligible (i.e., the effect of other factors is cancelled when that of a particular factor is considered). Here, the effect of nine factors (e.g., incubation time, pH, temperature, glycerol, glucose, starch, agitation speed, inoculums size, and yeast extract) on bacteriocin production was studied in twelve experimental trials. Each factor was examined in two levels designated as −1 (low level) and +1 (high level). The design relies on the fact that each factor is studied in a low level in a set of (*N* + 1)/2 trials and in a high level in the other set (*N* + 1)/2 trials. All trials were carried out in triplicate in 250 mL Erlenmeyer flasks with a working volume of 100 mL broth. The averages of three readings were taken as the experimental response value. PBD is based on a first order polynomial equation:

(1) $$\begin{array}{c} Y=\beta o+\Sigma \beta i\;xi, \end{array}$$

where *Y* is the response, *βo* is the model intercept, *xi* is the independent variable (factor), and *βi* is the independent variable estimate.

#### 2.7.2. Response Surface Methodology (RSM)

Response surface methodology approach in the form of Box-Behnken design (BBD) [23] was applied in this study to determine the optimal level of each key determinant that was identified by PBD. All forms of interactions among the independent variables are shown in this design. Each factor was deliberated in three levels denoted as −1 (low level), 0 (center level), and +1 (high level) through fifteen experimental runs. All trials were conducted in triplicate in 250 mL Erlenmeyer flasks with a working volume of 100 mL broth. The averages of three readings were considered as the response experimental values. Box-Behnken design (BBD) is based on a second order polynomial equation:

(2) $$\begin{array}{c} Y=\beta o+\beta n\;Xn+\Sigma \beta nn\;Xn\cdot Xn+\Sigma \beta nm\;Xn\cdot Xm, \end{array}$$

where *Y* is the response, *βo* is the model intercept, *βn* is the linear estimate, *βnn* is the quadratic estimate, and *βnm* is the interaction estimate.

### 2.8. Statistical Analysis of Data

PBD and BBD were created by Minitab, version 15.0. Multiple regressions, canonical analysis, and three-dimensional surface plots were performed via RSM package (R development Core team 2009), available from the Comprehensive R Archive Network (http://CRAN.R-project.org/package=rsm) [24].

### 2.9. Bacteriocin Bioassay and Its Inhibition Spectrum

The antimicrobial effect of bacteriocin against the abovementioned indicator strains was estimated using well cut diffusion assay. Briefly, 100 mL agar medium specific for each indicator strain (bacteria or yeast) separately was seeded with 1 mL of an overnight grown culture. For fungal strains, 1 mL of fungal spore suspension was used to inoculate 100 mL agar medium specific for fungi. After that, the seeded medium was poured into plates. After solidification, wells were created in plates. One hundred microliters of the bacteriocin producing culture was centrifuged at 8,000 rpm for 3 min (Mikro 200 Hettich, Germany). Each well was injected with 100 *μ*L of the cell-free supernatant containing the crude bacteriocin. The plates were incubated at 37°C for 1–3 days. The activity of the examined bacteriocin was expressed in arbitrary absolute units (AU). One AU is defined as inhibition zone diameter in mm produced by the tested bacteriocin on the bacterial indicator lawn [25]. AU/mL = inhibition diameter zone in mm·1000/volume of cell-free supernatant in *μ*L. Here, the bacterial indicator lawn involved in assessment of bacteriocin activity was* E. amylovora*.

### 2.10. Partial Characterization of Bacteriocin

The effect of different pH(s) and temperatures on the activity of the produced bacteriocin was examined. Moreover, the influence of some organic solvents (e.g., acetone, acetonitrile, toluene, methyl alcohol, and ethyl alcohol) on the activity of the tested bacteriocin was also studied. Furthermore, the impact of proteinase K on bacteriocin activity was examined to prove the proteinaceous nature of the claimed bacteriocin substance.

### 2.11. Monitoring the Production of Bacteriocin

Production of bacteriocin was monitored along with the growth of the producer isolate. Briefly, 100 mL of BYP broth was inoculated with 0.5 mL of an overnight grown culture. The newly inoculated broth was incubated at 37°C with agitation speed of 200 rpm (New Brunswick Incubator Shaker, USA). One mL samples were withdrawn from the culture each hour to measure the bacterial growth spectrophotometrically at 420 nm (UV-Vis Spectronic helios spectrophotometer, Thermo Scientific Co., USA). Then, the cell-free supernatants obtained via centrifugation at 7,000 rpm for 3 min (Mikro 200 Hettich, Germany) were kept at 4°C until their usage in well cut diffusion assay to determine the bacteriocin activity as mentioned above in Section 2.9.

## 3. Results

### 3.1. *Bacillus* sp. YAS 1 Strain

**Figure 1:**
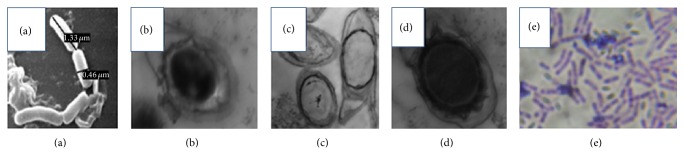
Electron micrographs ((a) SEM and (b)–(d) TEM) and (e) light micrograph of* Bacillus* sp. YAS 1 strain.

**Figure 2:**
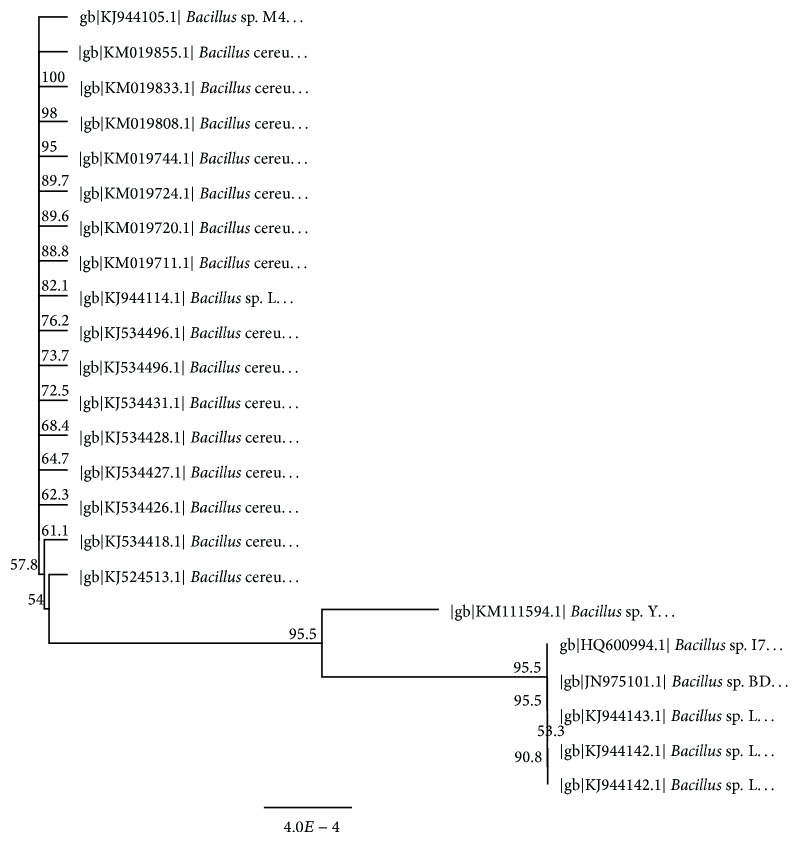
Neighbor-joining tree showing the phylogenetic relationship between 16S rDNA sequence of the candidate bacterial isolate S9 (taxonomically nominated as* Bacillus* sp. YAS 1 strain) and other 16S rDNA sequences belonging to closely related bacteria. Phylogenetic tree was constructed via Geneious R8 software. Numbers on branch nodes represent bootstrap values (1000 resamplings).

In the course of a screening programme for bacteriocin producers, eighty-seven samples (22 of fruits (F), 15 of leaves (L), and 50 of soils (S)) from different sites naturally infected with the pear fire blight disease were collected to isolate bacteriocin producing bacteria. Preliminary screening implicated only six bacterial isolates, nominated as F2, L18, S4, S9, S25, and S33, proving their capabilities for bacteriocin production through tooth picking technique and well diffusion assay versus the pear fire blight pathogen* E. amylovora* as shown in Table 1. The largest zone of inhibition (22.8 ± 2.5) was achieved upon using the antimicrobial agent of the isolate S9. The most potent producer (S9) was selected to conduct the current study. Morphological and biochemical identifications of the bacteriocin producer unraveled that this isolate was assigned to* Bacillus* sp. (Table 2 and Figure 1). Furthermore, this identification was verified via alignment of 16S rDNA sequence of the isolate S9 using BLASTN algorithm. Results of BLASTN demonstrated that the query sequence has 99% identity and 100% query coverage with the 16S rDNA of the bacterium recorded in the GenBank under the accession number HQ600994.1. Phylogenetic tree illustrated in Figure 2 inferred the degree of relatedness between 16S rDNA sequence of the isolate S9 with other closely related 16S rDNA sequences deposited in the international nucleotide sequences databases (e.g., GenBank, EMBL, DDBJ, etc.). The biochemical taxonomy for the bacterial isolate (S9) was in a good agreement with the molecular taxonomy. Based on these data, our bacterial isolate (S9) was nominated as* Bacillus* sp. YAS 1 strain. Additionally, the nucleotide sequence of* 16S rDNA gene* of* Bacillus* sp. YAS 1 strain was submitted in the GenBank and was given an accession number KM111594.

### 3.2. Optimizing of Bacteriocin (BAC YAS 1) Production

The bacteriocin produced from* Bacillus* sp. YAS 1 strain was nominated as BAC YAS 1. Its production was optimized through a two-step optimization procedure.

#### 3.2.1. PBD Results

**Figure 3:**
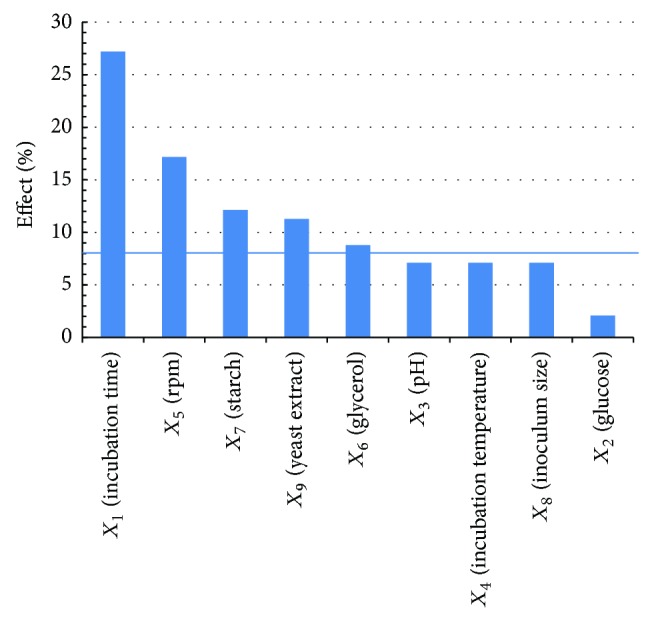
Pareto chart in a descending layout for Plackett-Burman parameter estimates of nine tested independent variables.

The matrix of PBD and the experimental values versus the predicted values of the process outcome were displayed in Table 3(a), whilst coded real values of the independent variables and results of linear multiple regression were illustrated in Table 3(b). The *F* and *P* values of the model were 57.58 and 0.017, respectively, as elucidated from ANOVA results. Significance of the model was indicated by this *F* value, while the model *P* value reflects that the chance is only 1.7% that this model *F* value could occur due to noise. Significance of coefficients was evaluated by looking at *P* value of the estimate. As a rule of thumb, significance of an estimate is inversely proportional to its *P* value and directly proportional to its *t*-test [26]. Based on data derived from linear multiple regression, there exist five independent variables (incubation time, agitation speed, glycerol, starch, and yeast extract) provoking significant consequences on BAC YAS 1 productivity. Only three (*X*_1_: incubation time, *X*_5_: agitation speed, and *X*_9_: yeast extract) out of the five independent variables imposed positive significance on the process outcome, whilst the other two remaining independent variables (*X*_6_: glycerol and *X*_7_: starch) showed dramatic negative significant impact. Pareto chart was applied here as an informative tool to display the significance consequences of the nine investigated independent variables on the process outcome in a descending order according to their *P* values (Figure 3). After exclusion of the non-significant model terms, a first order polynomial equation in terms of coded values was established to explain the linear relation between the independent variables and the process outcome as follows:

(3) Y=335.8+54.16X1+34.17X5 −17.5X6−24.16X7+22.5X9.

Independent variables demonstrating non-significant impact on the process outcome were either used in the medium at their lowest values (*X*_3_: pH, *X*_4_: incubation temperature, and *X*_8_: inoculums size) or excluded from the medium (*X*_2_: glucose) in the next experiments. On the contrary, independent variables (*X*_6_: glycerol and *X*_7_: starch) presenting dramatic negative significant effects were omitted from the production medium in the next experiments. However, incubation time (*X*_1_), agitation speed (*X*_5_), and yeast extract (*X*_9_) were identified as three key determinants controlling BAC YAS 1 productivity and were further subjected to detailed analysis through RSM approach.

#### 3.2.2. RSM Results

RSM is an empirical mathematical tool used in optimization processes purposes in order to locate the optimal level of a set of controllable factors along with the maximal level of the process outcome. Here, Box-Behnken design was applied. The design matrix and the experimental versus the predicted levels of BAC YAS 1 were shown in Table 4(a). Coded real values of the tested independent variables and results of multiple non-linear regression were displayed in Table 4(b). ANOVA results inferred that the model *F* value and *P* value were 9.99 and 0.01, respectively. These values implied the significance of the model and the chance (1%) that this *F* value could occur due to noise. Model aptness is evidenced by the value of *R* ^2^ (0.95) that conferred the good agreement between the experimental and the predicted values of the response. A second order polynomial equation was fixed in terms of coded values to explain all possible interactions among the tested independent variables that could impose an effect on the response:

(4) Y=443.3+25X1+31.25X5−8.75X9−21.66X1·X1 −94.16X5·X5−134.17X9X9−32.5X1·X5 +42.5X1·X9+5X5·X9.

Anchored in results of multiple non-linear regression only two model terms displayed significant effect (*P* < 0.05) on BAC YAS 1 productivity. There exist quadratic effects imposed by agitation speed and yeast extract on the process outcome.

To attain the optimized conditions, canonical analysis was performed. Determining the overall of the response shape and the nature of the stationary point whether it is maximum, saddle, or minimum could be achieved via canonical analysis. Eigenvalues and eigenvectors in the matrix of second order are used to characterize the response shape. Eigenvectors are used to determine the directions of principle orientation for the surface. However, signs and magnitude of eigenvalues indicate surface shape in these directions. Meyer 1976 stated two rules concerning the concept of eigenvalues and their mathematical designations [27]. Upward and downward curvatures are proved by positive and negative eigenvalues, respectively (1st rule). The larger an eigenvalue is in its absolute value, the more pronounced is the curvature of the response in the associated direction (2nd rule). Our model indicated that it has the eigenvalues (*λ*_1_= −1.44, *λ*_5_ = −9.76, and *λ*_9_ = −13.81). Based on 1st rule of Meyer, the present negative eigenvalues conferred that the stationary point is maximum. The two largest eigenvalues in their absolute values (*λ*_5_ = 9.76 and *λ*_9_ = 13.81) reflected the pronounced curvature of the response in the directions of the independent variables (*X*_5_ and *X*_9_). This finding authenticated to a great extent the results of regression analysis that stated that *X*_5_ and *X*_9_ showed the highest significant effect in quadratic forms on the level of BAC YAS 1. The predicted stationary point as derived from canonical analysis along with differentiation of (4) was found to be at coded values {*X*_1_ = 0.59, *X*_5_ = 0.06 and *X*_9_ = −0.059} to achieve a predicted level of BAC YAS 1 of 470 AU/mL. This predicted stationary point is obviously located inside the model domain (model constrains).

**Figure 4:**
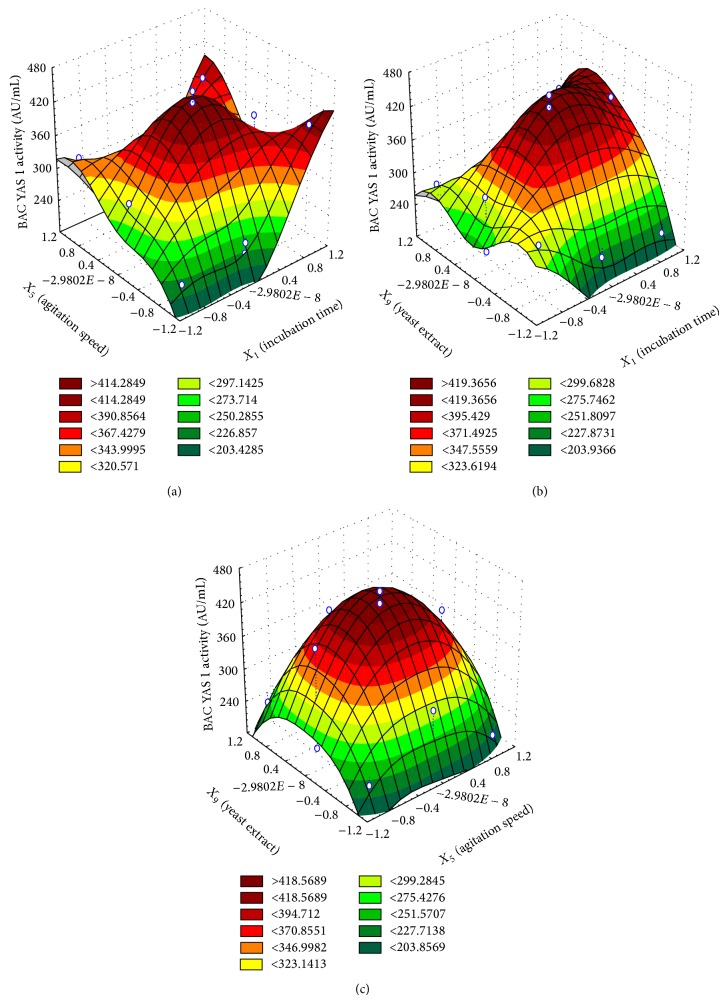
(a) Three-dimensional surface plot for the dependent variable BAC YAS 1 antimicrobial activity versus the independent variables incubation time and agitation speed. (b) Three-dimensional surface plot for the dependent variable BAC YAS 1 antimicrobial activity versus the independent variables incubation time and yeast extract. (c) Three-dimensional surface plot for the dependent variable BAC YAS 1 antimicrobial activity versus the independent variables agitation speed and yeast extract.

Three-dimensional surface plots were depicted to further explore the nature of the response at the stationary point as shown in Figures 4(a), 4(b), and 4(c). Normally, the three-dimensional surface plots are based on the model, keeping one independent variable constant at its optimal level where changing the other two independent variables within the design constrains.

Figure 4(a) illustrated the response of the dependent variable (BAC YAS 1) for the optimal level of the independent variable yeast extract. The maximal predicted level of BAC YAS 1 (470 AU/mL) was noticed at levels of 62 hrs and 207 rpm. Supportively, the three-dimensional surface plot depicted in Figure 4(b) revealed that the maximal point of BAC YAS 1 at the optimal level for agitation speed could be reached at 62 hrs and 0.48% (w/v) yeast extract. Likewise, these predicted levels of both dependent and the independent variables were further proved by the three-dimensional surface plot illustrated in Figure 4(c).

#### 3.2.3. Model Validation

**Figure 5:**
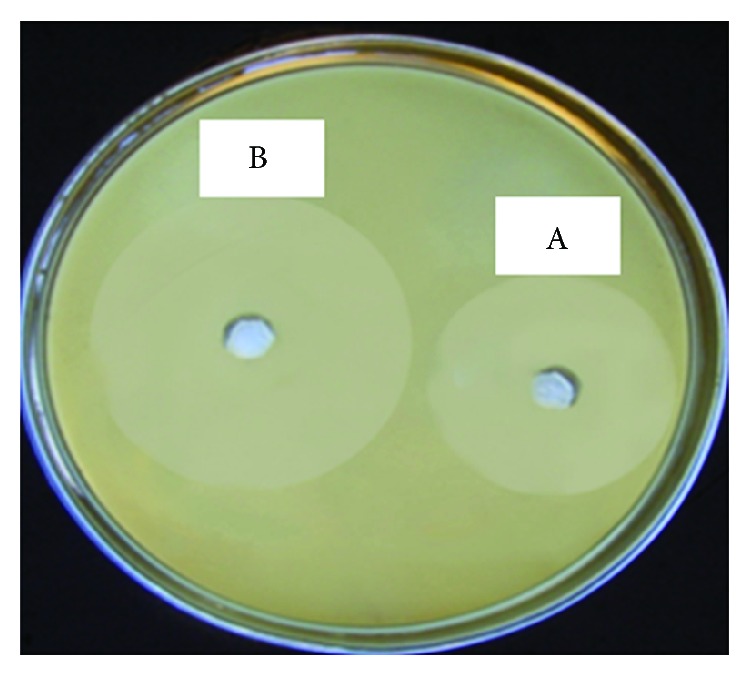
Antimicrobial activity of BACYAS 1 on* E. amylovora* upon using non-optimized conditions (A) and optimized conditions (B).

In order to determine model adequacy, the predicted levels of incubation time, agitation speed, and yeast extract were fully considered in the laboratory. The experiment was run in triplicate. Data indicated that the maximal obtained level of BAC YAS 1 was 470 (AU/mL). The obtained data conferred the high precision of model 100% (i.e., good agreement between the predicted and the experimental levels of BAC YAS 1). Furthermore, by the end of the optimization plan, the level of BAC YAS 1 was enhanced 1.6-fold that was obtained upon using the non-optimized medium as shown in Figure 5.

### 3.3. BAC YAS 1 Production Time Course versus Bacterial Growth

**Figure 6:**
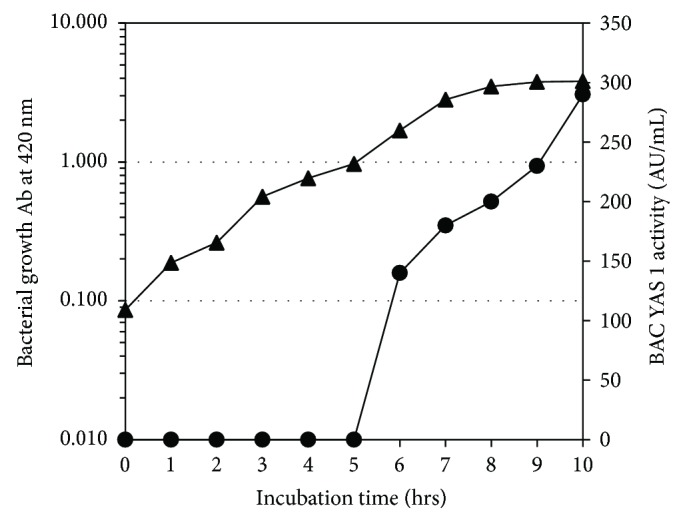
The BAC YAS 1 production time course vs. the growth of* Bacillus* sp. YAS 1 strain on bacteriocin optimized medium. Symbol ▲: bacterial growth. Symbol ●: BAC YAS 1 activity (AU/mL) upon using the indicator strain* E. amylovora*.

Production of BAC YAS 1 during the growth of* Bacillus* sp. YAS 1 strain was monitored.  Figure 6 demonstrated that the first onset of BAC YAS 1 was observed after five hrs of the bacterial growth (i.e., late exponential phase). Increased levels of BAC YAS 1 were recorded during the stationary phase of bacterial growth.

### 3.4. Partial Characterization of BAC YAS 1

The antimicrobial activity of the crude BAC YAS 1 was completely lost after treatment with proteinase K (data not shown). Total inactivation of the antimicrobial activity by proteinase K greatly affirms the proteinaceous nature of BAC YAS 1. On the other hand, BAC YAS 1 could maintain its full antimicrobial activity over a wide range of pH (3–12). A dramatic decrease in BAC YAS 1 antimicrobial activity was noticed only at the extreme acidic or alkaline pH(s) (1, 2, and 13). Additionally, present data revealed a good stability of BAC YAS 1 over a wide range of pH (1–13) along five hrs of incubation at the indicated pH(s). Pertaining to the effect of temperature on antimicrobial activity of BAC YAS 1 (Table 5), full activity of BAC YAS 1 was detected after 15 min of exposure at 60°C, whilst BAC YAS 1 could preserve 83% of its activity at 75°C after 15 min of heat exposure. A dramatic decrease in BAC YAS 1 activity was observed after exposure to temperatures greater than 75°C. Complete loss of activity was noted at 85°C. Concerning the effect of some organic solvents on BAC YAS 1, none of the tested organic solvents stated an inhibitory effect on the BAC YAS 1 activity.

### 3.5. Antimicrobial Spectrum of BAC YAS 1

BAC YAS 1 offered antimicrobial activity against some Gram-negative human pathogens (e.g.,* S. typhimurium, E. aerogenes*,* Proteus* sp.,* Klebsiella* sp.,* Enterococcus* sp., and* C. jejuni*), Gram-positive human pathogens (e.g.,* C. perfringens* and* S. epidermidis*), a food spoilage bacterium (e.g.,* L. innocua*), and a plant pathogen bacterium (*E. amylovora*) as well, while the growth of other human pathogens (*S. aureus*,* S. typhi*,* S. paratyphi,* and* S. boydii*) had not been affected by BAC YAS 1 antimicrobial activity. Among the examined indicator strains, the most sensitive strains to BAC YAS 1 could be listed in the following order regarding the inhibition zone diameter:* E. amylovora*,* L. innocua*,* Klebsiella* sp., and* S. epidermidis*. Additionally, no antimicrobial activity of BAC YAS 1 could be detected against* E. coli* and* E. faecalis*. Regarding the influence of BAC YAS 1 on some environmental bacterial strains, the growth of only two strains belonging to the genus* Bacillus* (*B. licheniformis* SHG2 and* B. subtilis* AS1) was inhibited by BAC YAS1. Conversely, none of the tested fungal and yeast strains were influenced by BAC YAS 1 antimicrobial activity. Surprisingly, none of the tested lactic acid bacterial strains (*Lactobacillus bulgaricus*,* L. casei*,* L*.* lactis,* and* L. reuteri*) were affected by BAC YAS 1 antimicrobial activity as shown in Table 6.

## 4. Discussion

Although the literature reported the production of bacteriocins from bacteria belonging to different genera and species such as lactic acid bacteria,* Bacillus* spp.,* Escherichia coli*, and* Vibrio* spp., a few reports exist concerning the key determinants controlling the levels of the produced bacteriocins from these bacteria. Additionally, the bottleneck for bacteriocin production from most bacterial strains is the low yield. In this context, optimizing BAC YAS 1 production is the first step in the agenda of large scale production. Here, the production of BAC YAS 1 was affected by neither soluble starch nor initial pH of the production medium. The present finding is inconsistent with other findings of Zhong et al. [28], Pal et al. [29], and Kaur et al. [30], where the production of bacteriocin from* B. cereus* XH25,* Weissella paramesenteroides* DFR-8, and* Pediococcus acidilactici* BA28, respectively, was significantly influenced by soluble starch and initial pH of the production medium. However, present finding concerning the impact of yeast extract on BAC YAS1 was in good agreement with those of Zhong et al. and Meera and Devi who stated that yeast extract did exert significant effect on the productivity of a bacteriocin-like substance from* B. cereus* XH25 and probiotic lactic acid bacteria, respectively [28, 31]. Pertaining to the influence of glycerol on BAC YAS 1 productivity, it did not exhibit any significant consequence. The present finding was in discordance with those of Yi et al. [32] and Kamoun et al. [33] where addition of glycerol resulted in an increase in the productivity of bacteriocin from* Lactobacillus paracasei* J23 and Bacthuricin F4 (bacteriocin) produced by* B. thuringiensis* subsp.* kurstaki* strain, respectively. Glucose was one of the noninfluential factors exerted on productivity of BAC YAS 1, whilst glucose exerted a significant impact on the productivity bacteriocin from* W. paramesenteroides* DFR-8 [29],* Bacillus* sp. GU057 [34], and Bacthuricin F4 (bacteriocin) produced by* B. thuringiensis* subsp.* kurstaki* strain [33]. With respect to optimal temperature for bacteriocin production, there exists a discrepancy in optimal temperature to produce bacteriocin from various bacteria [35–37]. The observed discrepancy in key determinants controlling the productivity of various bacteriocins produced by different bacteria greatly addresses the indispensable need for carrying out optimization step for each newly isolated bacteriocin producer. Alleviation of the overall cost for a bioprocess commercialization is considered one of the great challenges. Nonetheless, the overall cost of a bioprocess is usually constricted to the high cost of production medium. BAC YAS 1 production medium is somehow simple and cost effective upon comparison with other media required for bacteriocins production from other bacterial sources (e.g., MRS medium plus other supplements required for bacteriocin production from lactic acid bacteria). Therefore, the cost encountered in BAC YAS1 production will alleviate. This in turn gives BAC YAS 1 productivity from* Bacillus* sp. YAS 1 a privilege over productivity of other bacteriocins from other bacteria. Hence, the overall cost for BAC YAS 1 production will reduce. From another side, the timeline for BAC YAS 1 production from* Bacillus* sp. YAS1 strain is 62 hrs which is to some extent not a prolonged time. Subsequently, this will create a rapid process upon scaling up the production of BAC YAS1.

Determining the onset of the genes encoding for bacteriocin is one of the prerequisite data in the agenda of bacteriocin production on an industrial scale. Thus, BAC YAS 1 production was monitored along with the growth of its producer strain* Bacillus* sp. YAS 1 in the bacteriocin production medium. The onset of BAC YAS 1 production was recorded to be at the late exponential phase and increased during the stationary phase of growth reaching its maximum after 62 hrs of cultivation. This finding was in disagreement with other reported findings [29, 38–41]. For instance, the production of bacteriocin (Bac14) by* B. subtilis* 14B started after a 24-h-lag phase and then increased exponentially and reached its maximum within 96 h cultivation [38]. BAC YAS 1 production was not associated with the growth of* Bacillus* sp. YAS 1 strain because the maximum yield of BAC YAS 1 was achieved during the stationary phase. The present finding is partially in agreement with that of cerein from* B. cereus* where cerein could be traced at the beginning of stationary phase [1, 39]. On the other hand, the present finding was contradicted by Pal et al. and Banerjee et al. who affirmed that the bacteriocin of* W. paramesenteroides* DFR-8 [29] and that of* L. brevis* FPTLB3 [40] were growth-dependent since majority of the bacteriocin was traced during the exponential phase of growth. Activity of cerein 7 was detected in cultures of* B. cereus* Bc7 near the end of exponential phase and reached its maximum at the beginning of stationary phase [41]. It seems that the onset of bacteriocins encoding genes varies widely not only among distally related species and genera but also among strains belonging to the same species which necessitates the indispensable need to explore the exact time at which each bacteriocin encoding gene turns on in each newly isolated producer strain.

To gain more insights into BAC YAS 1, partial characterization was performed as mentioned above. Bacteriocins by definitions are sensitive to the action of proteolytic enzymes. Sensitivity of BAC YAS 1 to the action of proteinase K confers its proteinaceous nature and agrees well with the reported sensitivity of other bacteriocins to proteinase K [26, 40, 42, 43]. In contrast, the temperature stability profile for various bacteriocins [39, 42, 44] was somehow comparable to that of BAC YAS 1. For instance, the temperature stability profile (45°C–75°C) for cerein 8A and cerein of* B. cereus* [1, 5] was in partial agreement with that of the present finding. Conversely, Bac14B of* B. subtilis* [38] exerted complete higher thermostability over a broad range (30°C–100°C) for two hrs when compared to that of BAC YAS 1 over a broad range (45°C–80°C) for 15 min. Moreover, bacteriocin from* Propionibacterium thoeniit* was found to be heat labile [45]. Regarding optimal pH and pH stability of BAC YAS 1, present findings were somehow consistent with some reported bacteriocins derived from either closely related bacterial genera or other bacteriocins from other distally related bacterial genera and/or species [1, 31, 39, 42, 45]. For example, Naclerio et al. [39] stated that cerein of* B. cereus* exhibited pH stability profile (3–12) quite similar to that of BAC YAS 1. Nevertheless, Kayalvizhi and Gunasekaran reported a pH stability profile (3–10) of bacteriocin from* B. licheniformis* MKU3 [42]. Bacteriocins of* B. subtilis* 14B and* L. brevis* FPTLB3 demonstrated lower pH stability range (3–8) [38, 40]. This reported divergence in thermostability and pH stability broadened by bacteriocins from different bacteria is mainly attributed to their amino acids structures that necessarily impose a strong influence on their physicochemical properties. This highlights the substantial necessity to unravel the amino acid structure of the BAC YAS 1 under study. Further characterization of BAC YAS 1 implied its ability to retain the full activity in presence of some tested organic solvents (e.g., chloroform, acetone, ethyl alcohol, acetonitrile, and methyl alcohol). Sensitivity of bacteriocin to organic solvents differs among different bacteriocins from different bacteria [1, 38, 39, 42]. This retained activity in presence of the abovementioned organic solvent may give a hint about the hydrophobic nature of BAC YAS 1. Additionally, retained activity of BAC YAS 1 under a wide range of pH could be attributed to the type of amino acid residues of BAC YAS 1. Consequently these two findings profoundly address the indispensable need to study the amino acid composition of BAC YAS 1 in the future.

Bacteriocins by definitions are antimicrobial agents and those that demonstrate wide spectrum antimicrobial activity against both Gram-negative and Gram-positive bacteria are of a prime importance as chemotherapeutic and prophylactic agents. BAC YAS 1 demonstrated a broad spectrum antimicrobial activity against both Gram-negative and Gram-positive bacteria as mentioned above. The antimicrobial spectrum activity of BAC YAS 1 exhibited a similar pattern to that obtained by bacteriocins from the lactic acid bacteria (*L. plantarum* and* L. fermentum*) where their bacteriocins exerted a profound antimicrobial effect against some Gram-positive bacteria (e.g.,* S. aureus* and* Streptococcus pneumoniae*) and Gram-negative bacteria (e.g.,* E. coli*,* P. aeruginosa*,* Klebsiella,* and* Proteus*) [46]. In contrast, the BAC YAS 1 antimicrobial spectrum did not agree with other bacteriocins [41, 47]. Bacteriocins of* B. cereus* Bc7 and lactic acid bacteria (e.g.,* L. lactis* and* P. acidilactici*) inhibited a broad spectrum of Gram-positive bacteria, including food-borne pathogens but not any of the tested Gram-negative bacteria [41, 47]. In this context, cerein of* B. cereus* displayed antimicrobial effect against only closely related* Bacillus* spp. [1]. On top and above, the tested lactic acid bacterial species have not been influenced by the antimicrobial activity of BAC YAS 1. This finding in turn gives BAC YAS 1 a privilege over other bacteriocins reported in the literature and underpins its potential usage as a food biopreservative without killing the beneficial lactic acid bacteria included in foodstuffs.

## 5. Conclusion

In a nutshell, employment of optimization strategies in bioprocesses particularly for bacteriocin industry is considered to be an essential step. It aims not only to minimize the cost encountered in medium compositions but also to maximize the yield as well. By the end of sequentially statistical optimization procedure, we could end up with appreciable levels of the bacteriocin (470 AU/mL) after 62 hrs of the growth of* Bacillus* sp. YAS 1 strain under optimized conditions against* E. amylovora*. Conclusively, BAC YAS 1 of* Bacillus* sp. YAS 1 strain is a promising bacteriocin that could offer numerous biotechnological applications in wide variety of fields: agricultural, medical, and industrial ones as a biocontrol agent against the fire blight plant pathogen* E. amylovora*, as an antibiotic agent against the human pathogens (e.g.,* C. perfringens*,* S. epidermidis*,* S. typhimurium*,* E. aerogenes*,* C. jejuni*,* Enterococcus* sp.,* Proteus* sp., and* Klebsiella* sp.) and as a food biopreservative against the food spoiler* L. innocua*. On top and above, absence of BAC YAS 1 inhibitory effect towards the beneficial lactic acid bacteria along with its potent inhibitory effect against the food spoiler* L. innocua* donates BAC YAS 1 a privilege over other bacteriocins derived from other bacteria.

The present data exceedingly prompt applying directed evolution methodologies in order to improve the characteristics of the promising broad spectrum antimicrobial activity of BAC YAS 1 in the future work. In this context, improving thermostability of the BAC YAS 1 over a wide range of temperature degrees along with prolonged exposure times is an issue of a prime importance if commercial industrialization of BAC YAS 1 is being intended in the future. Rational protein design, one of the currently applied methodologies, could be a powerful tool to achieve this task that in turn necessitates the indispensable need to purify and study the crystalline structure of BAC YAS 1.

## Figures and Tables

**Table 1:** Sensitivity of *E*. *amylovora* to antimicrobial agents produced by six bacterial isolates.

Bacterial isolate						
**Screening method**	**F2**	**L18**	**S4**	**S9**	**S25**	**S33**
*Toothpick technique* (mm)^a^	13.0	7.0	7.7	14.3	4.0	17.3
*Well cut diffusion method* (mm^b,c^ ± SE^d^)	6.3 ± 1.17	Nil	19.3 ± 1.8	**22.8 ± 2.5**	7.7 ± 1.8	15.6 ± 1.88

^a,b^mm = diameter of inhibition zone; ^c^mean of three readings; ^d^standard error.

**Table 2:** Morphological and biochemical identification of S9 bacteriocin producing bacterial isolate.

Test	Result
Cell shape	Rods
Gram stain	+ve
Spore formation	+ve
Growth on MYP	Non-mannitol utilizer
Motility	+ve
Growth at pH 6–8	+ve
Growth at 50°C	+ve
Growth at 7.5% NaCl	−ve
Starch hydrolysis	**+ve**
Casein hydrolysis	+ve
Tween 20 hydrolysis	−ve
Nitrate reduction	**+ve**
Catalase	+ve
Oxidase	+ve
Urease	−ve
Indole production	−ve
Citrate utilization	−ve
Voges-Proskauer (VP)	**+ve**
Methyl red (MR)	−ve
Gelatin liquefaction	+ve
Hemolysis on sheep blood agar	*β*-Hemolytic
Lecithinase on MYP	+ve
Penicillin resistance	+ve
Oxidative fermentation (OF):	
Glucose fermentation	+ve (yellow/blue: O/NF)^*^
Galactose fermentation	+ve (yellow/blue: O/NF)
Sucrose fermentation	+ve (yellow/yellow: O/F)^**^

^*^O/NF: oxidative/non-fermentative.

^**^O/F: oxidative/fermentative.

**(a):** 

Trial number	*X*_1_	*X*_2_	*X*_3_	*X*_4_	*X*_5_	*X*_6_	*X*_7_	*X*_8_	*X*_9_	*Y* (AU/mL CFS)^*^	
										Exp.^a^	Pred.^b^
1	−1	−1	1	1	1	−1	1	1	−1	290	296.67
2	−1	−1	−1	−1	−1	−1	−1	−1	−1	250	248.33
3	−1	1	1	1	−1	1	1	−1	1	220	218.33
4	−1	1	1	−1	1	−1	−1	−1	1	340	341.67
5	1	−1	−1	−1	1	1	1	−1	1	380	386.67
6	1	1	−1	1	1	−1	1	−1	−1	420	413.33
7	1	−1	1	−1	−1	−1	1	1	1	360	353.33
8	−1	−1	−1	1	1	1	−1	1	1	390	383.33
9	1	−1	1	1	−1	1	−1	−1	−1	320	321.67
10	1	1	−1	1	−1	−1	−1	1	1	460	466.67
11	1	1	1	−1	1	1	−1	1	−1	400	398.33
12	−1	1	−1	−1	−1	1	1	1	−1	200	201.67

^a^Experimental values and  ^b^predicted values [*X*_1_: incubation time, *X*_2_: glucose, *X*_3_: pH, *X*_4_: incubation temperature, *X*_5_: agitation speed, *X*_6_: glycerol, *X*_7_: starch, *X*_8_: inoculum size, and *X*_9_: yeast extract]; ^*^AU/mL sample: arbitrary units as a measure for bacteriocin activity = diameter of inhibition zone in mm. 1000/volume of CFS in *μ*L.

^*^CFS: cell-free supernatant.

**(b):** 

Independent variable	Low level	High level	Main effect	*B*-coefficient	*P* value	*t*-value	% confidence
	−1	+1					
**Incubation time** (**X**_1_, **h** **r** **s**)	24	48	108.34	54.17	**0.004** ^*^	15.76482	**99.6** ^*^
Glucose (*X*_2_, w/v%)	1	2	8.33	4.167	0.349	1.212678	65.1
pH (*X*_3_)	6.5	7.5	−28.33	−14.167	0.0541	−4.12311	94.59
Incubation temperature (*X*_4_, °C)	28	30	28.33	14.167	0.0541	4.123106	94.59
**Agitation speed** (**X**_5_, **r** **p** **m**)	120	200	68.33	34.167	**0.0099** ^*^	9.943961	**99.01** ^*^
**Glycerol** (**X**_6_, **w**/**v**%)	0	1	−35.00	−17.5	**0.0363** ^*^	−5.09325	**96.37** ^*^
**Starch** (**X**_7_, **w**/**v**%)	0	1	−48.33	−24.167	**0.0196** ^*^	−7.03353	**98.04** ^*^
Inoculum size (*X*_8_, v/v%)	10	12	28.33	14.167	0.054095	4.123106	94.59
**Yeast extract** (**X**_9_, **g**/**L**)	3	5	45.00	22.500	**0.0225** ^*^	6.548462	**97.75** ^*^

^*^Significant *P* value <0.05; *R* ^2^ = 0.99; adjusted *R* ^2^ = 0.98; and *P* value for the model = 0.017.

**(a):** 

Trial number	*X*_1_	*X*_5_	*X*_9_	*Y* (AU/mL CFS)^*^	
				Exp.^a^	Pred.^b^
1	−1	−1	0	220	238.75
2	1	−1	0	400	366.25
3	−1	1	0	320	353.75
4	1	1	0	370	351.25
5	−1	0	−1	300	313.75
6	1	0	−1	210	211.25
7	−1	0	1	280	278.75
8	1	0	1	360	346.25
9	0	−1	−1	220	187.50
10	0	1	−1	200	180.00
11	0	−1	1	240	260.00
12	0	1	1	200	232.50
13	0	0	0	430	443.33
14	0	0	0	450	443.33
15	0	0	0	450	443.33

^a^Experimental values and  ^b^predicted values.

*X*_1_: incubation time, *X*_5_: agitation speed, and *X*_9_: yeast extract.

^*^AU/mL sample: arbitrary units as a measure for bacteriocin activity = diameter of inhibition zone in mm. 1000/volume of CFS in *μ*L.

^*^CFS: cell-free supernatant.

**(b):** 

Variable	Low level	Middle level	High level	Model term	Main effect	*B-*coefficient	*t*-value	*P* value	% confidence
	−1	0	+1						
Incubation time (*X*_1_, hrs)	24	48	72	*X*_1_	50.00	25	1.65144	0.159558	84.0
Agitation speed (*X*_5_, rpm)	80	200	320	*X*_5_	62.50	31.25	0.07506	0.943073	5.69
Yeast extract (*X*_9_, g/L)	2	5	8	*X*_9_	−17.50	−8.75	1.12598	0.311295	68.87
				*X*_1_ · *X*_1_	−43.34	−21.67	−0.88395	0.417196	58.28
				*X*_5_ · *X*_5_	−188.34	−94.17	−3.84178	**0.012102** ^*^	**98.79** ^*^
				*X*_9_ · *X*_9_	−268.34	−134.17	−5.47369	**0.002773** ^*^	**99.72** ^*^
				*X*_1_ · *X*_5_	−65.00	−32.50	−1.38007	0.226083	77.39
				*X*_1_ · *X*_9_	85.00	42.50	1.80470	0.130962	86.90
				*X*_5_ · *X*_9_	−10.00	−5.00	−0.21232	0.840243	15.98

^*^Significant *P* value <0.05; *R* ^2^ = 0.95; adjusted *R* ^2^ = 0.85; *P* value for the model = 0.01.

**Table 5:** Effect of temperature on BAC YAS 1 antimicrobial activity.

Temperature (°C)	Residual activity%^*^
Control (BAC YAS 1 without treatment)	100
45°C	100
60°C	100
75°C	83
80°C	36
85°C	0.0

^*^Residual activity = AU after BAC YAS 1 treatment/AU before BAC YAS 1 treatment.

Treatment: 15 min exposure to each indicated temperature.

**Table 6:** Sensitivity of some indicator strains to BAC YAS 1.

Indicator strain	BAC YAS 1 activity (inhibition zone diameter, mm ± SE)^*^	Gram nature
**Bacteria**		
*Acinetobacter *sp. (human clinical isolate, Department of Microbiology, Faculty of Medicine, University of Alexandria, Egypt)	0.00	Gram-negative
*Bacillus cereus* (environmental strain isolated from soil, Egypt)	0.00	Gram-positive
*B. licheniformis *SHG2 (environmental strain isolated from soil, Egypt)	**13 ± 1.70**	Gram-positive
*B. licheniformis *SHG6 (environmental strain isolated from soil, Egypt)	0.00	Gram-positive
*B. licheniformis *SHG10 (environmental strain isolated from soil, Egypt)	0.00	Gram-positive
*B. subtilis *EMBLAKE (environmental strain isolated from El-Mahmoudia Lake, Egypt)	0.00	Gram-positive
*B. subtilis *AS1 (environmental strain isolated from soil, Egypt)	**10.25 ± 0.48**	Gram-positive
*B. subtilis *NCTC 10400/ATCC 6633	0.00	Gram-positive
*B. subtilis *(environmental strain isolated from soil, Egypt)	0.00	Gram-positive
*Bacillus *sp. Ash2 (environmental strain isolated from soil, Egypt)	0.00	Gram-positive
***Campylobacter jejuni *NCTC 11322/ATCC 29428**	**14.00 ± 0.41**	Gram-negative
***Clostridium perfringens *NCTC 8237/ATCC 13124**	**15.50 ± 1.5**	Gram-positive
*Citrobacter *sp. (human clinical isolate, Department of Microbiology, Faculty of Medicine, University of Alexandria, Egypt)	0.00	Gram-negative
***Enterobacter aerogenes* NCTC10006/ATCC13043**	**15.50 ± 0.46**	Gram-negative
*Enterobacter *sp. (human clinical isolate I, Department of Microbiology, Faculty of Medicine, University of Alexandria, Egypt)	0.00	Gram-negative
*Enterobacter *sp. (human clinical isolate II, Department of Microbiology, Faculty of Medicine, University of Alexandria, Egypt)	0.00	Gram-negative
***Enterococcus *sp.** (human clinical isolate, Department of Microbiology, Faculty of Medicine, University of Alexandria, Egypt)	**13.75 ± 0.35**	Gram-negative
*E. coli *(human clinical isolate, Department of Microbiology, Faculty of Medicine, University of Alexandria, Egypt)	0.00	Gram-negative
*E. coli *NCTC 12241/ATCC 25922	0.00	Gram-negative
*Enterococcus faecalis *NCTC 12697/ATCC 29212	0.00	Gram-negative
***E. amylovora*** (Department of Plant Pathology, Faculty of Agriculture, University of Alexandria, Egypt)	**41.00 ± 0.43**	Gram-negative
***Klebsiella *sp.** (human clinical isolate, Department of Microbiology, Faculty of Medicine, University of Alexandria, Egypt)	**29.5 ± 0.4**	Gram-negative
*Lactobacillus bulgaricus *(dairy product isolate, Department of Dairy Products, Faculty of Agriculture, University of Alexandria, Egypt)	0.00	Gram-positive
*L. casei *(dairy product isolate, Department of Dairy Products, Faculty of Agriculture, University of Alexandria, Egypt)	0.00	Gram-positive
*L. lactis* (dairy product isolate, Department of Dairy Products, Faculty of Agriculture, University of Alexandria, Egypt)	0.00	Gram-positive
*L. reuteri* (dairy product isolate, Department of Dairy Products, Faculty of Agriculture, University of Alexandria, Egypt)	0.00	Gram-positive
***Listeria innocua *NCTC 11288/ATCC 33090**	**37.75 ± 0.49**	Gram-positive
*Pseudomonas aeruginosa* strain EGY II	0.00	Gram-negative
*P. aeruginosa* (human clinical isolate, Department of Microbiology, Faculty of Medicine, University of Alexandria, Egypt)	0.00	Gram-negative
***Proteus *sp**. (human clinical isolate, Department of Microbiology, Faculty of Medicine, University of Alexandria, Egypt)	**14.00 ± 0.3**	Gram-negative
***Salmonella typhimurium* NCTC 12023/ATCC 14028**	**17.25 ± 0.49**	Gram-negative
*S. typhi *(human clinical isolate, Department of Microbiology, Faculty of Medicine, University of Alexandria, Egypt)	0.00	Gram-negative
*S. paratyphi* (human clinical isolate, Department of Microbiology, Faculty of Medicine, University of Alexandria, Egypt)	0.00	Gram-negative
*Shigella boydii *ATCC 9207	0.00	Gram-negative
*Staphylococcus aureus *NCTC 12981/ATCC 25923	0.00	Gram-positive
***Staphylococcus epidermidis *NCTC 13360/ATCC 12228**	**23.75 ± 0.75**	Gram-positive
*Vibrio parahaemolyticus *NCTC 3178	0.00	Gram-negative
**Fungi and yeast**		
*Aspergillus brasiliensis *NCPF 2275/ATCC 16404	0.00	
*Candida albicans * NCPF 3179/ATCC 10231	0.00	
*C. tropicalis *(human clinical isolate from diabetic foot, Department of Microbiology, Medical Research Institute, University of Alexandria, Egypt)	0.00	
*Saccharomyces cerevisiae *NCPF 3178	0.00	

^*^Mean of four readings with standard error (SE).
